# From MRI-defined bone marrow lesions to pain, structural progression, and subchondral remodeling in knee osteoarthritis: an integrated multi-database bibliometric and text-mining analysis

**DOI:** 10.3389/fmed.2026.1903675

**Published:** 2026-10-09

**Authors:** Yousheng Zhang, Zihang Zhao

**Affiliations:** 1 Faculty of Medicine, Dentistry and Health Sciences, The University of Melbourne, Melbourne, VIC, Australia; 2 Department of Orthopaedic Surgery, The Third Hospital of Hebei Medical University, Shijiazhuang, China

**Keywords:** bibliometric analysis, bone marrow edema-like lesion, bone marrow lesion, co-citation analysis, knee osteoarthritis, magnetic resonance imaging, subchondral bone, text mining

## Abstract

**Objective:**

To characterize the publication landscape, intellectual structure, thematic evolution, and emerging clinical, mechanistic, and translational directions of magnetic resonance imaging (MRI)-defined bone marrow lesion (BML) research in knee osteoarthritis.

**Methods:**

Web of Science Core Collection, Scopus, and PubMed were searched using core concepts related to knee osteoarthritis, MRI, and BMLs or bone marrow edema-like lesions. The searches initially identified 2,452 records, of which 2,415 were exported after database-level filters. Following cross-database deduplication, eligibility assessment, and application of the prespecified publication cutoff, 726 publications constituted the final integrated corpus. Descriptive bibliometrics, collaboration-network analysis, reference co-citation analysis, keyword and MeSH mapping, temporal thematic analysis, term frequency-inverse document frequency-based document clustering, and dictionary-based bridge-theme mapping were performed. Candidate cluster solutions from k = 2 to k = 10 were evaluated using within-cluster sum of squares, silhouette width, gap statistics, and repeated-subsample stability.

**Results:**

Publication activity increased substantially from 1996 through 2025, with the partial 2026 count reflecting publications available by June 1. Osteoarthritis and Cartilage was the leading journal, and the United States, Australia, Germany, China, and the United Kingdom were the major contributing countries. The knowledge base was anchored by studies of whole-joint MRI assessment, BML-related pain, structural progression, and imaging-pathology interpretation. Six exploratory text-derived research domains were identified: post-traumatic and surgical BML outcomes; community-based structural and risk correlates; subchondral biology, biomarkers, and mechanisms; longitudinal whole-joint progression and symptoms; pharmacologic, biologic, and interventional treatment; and MRI quantification and methodological validation. No single diagnostic identified a uniquely optimal cluster number; the six-domain solution was retained as an interpretable intermediate-granularity representation with moderate resampling stability. Exclusion of the 28 publications dated 2026 did not materially alter the publication trajectory, leading recent terms, or temporal-theme prevalence.

**Conclusion:**

MRI-defined BML research in knee osteoarthritis has developed into a multidisciplinary field connecting imaging phenotypes with symptoms, structural progression, subchondral remodeling, biomechanics, inflammation, and treatment-oriented investigation. Future studies should integrate longitudinal lesion trajectories, quantitative imaging, adjacent joint pathology, biomechanical measures, biological markers, and pain phenotyping to clarify clinically meaningful BML subtypes and their therapeutic implications.

## 1 Introduction

Knee osteoarthritis is a major cause of chronic pain, impaired mobility, and disability worldwide, and its population burden is projected to increase substantially over the coming decades (1). Contemporary models no longer regard knee osteoarthritis as a disorder of cartilage wear alone. It is increasingly understood as a heterogeneous whole-joint disease involving cartilage, subchondral bone, synovium, menisci, ligaments, periarticular tissues, and pain-processing pathways (2, 3). This broader framework is clinically important because symptoms, function, and structural progression correlate only partly with radiographic joint-space narrowing or cartilage loss.

Magnetic resonance imaging (MRI) has been central to this transition because it enables multiple joint tissues to be evaluated within the same examination. Among MRI-defined abnormalities, bone marrow lesions (BMLs), also described as bone marrow edema-like lesions, have attracted sustained interest. Contemporary reviews describe BMLs as subchondral imaging phenotypes associated with pain, structural progression, local biomechanics, bone-cartilage interactions, and treatment response (4, 5). These interpretations build on foundational studies linking BMLs with knee pain and osteoarthritis progression (6, 7). Imaging-pathology correlation has further shown that the edema-like MRI signal may reflect heterogeneous tissue changes, including trabecular abnormalities, fibrosis, necrosis, vascular alterations, microdamage, and remodeling, rather than simple fluid accumulation (8).

The clinical interpretation of BMLs remains difficult because they are measurable imaging features but not uniform pathological entities. The implications of a lesion may depend on its size, location, persistence, compartmental context, adjacent cartilage or meniscal damage, synovitis or effusion, local loading, and the patient's pain phenotype. Semi-quantitative whole-joint MRI scoring systems, including the Whole-Organ Magnetic Resonance Imaging Score and the MRI Osteoarthritis Knee Score, were therefore important not only as measurement tools but also as conceptual frameworks. They enabled BMLs to be evaluated together with cartilage defects, osteophytes, meniscal lesions, synovitis, effusion, and other osteoarthritis-related features (9, 10). BMLs consequently became quantifiable components of whole-joint phenotyping rather than isolated marrow signal abnormalities.

Despite extensive clinical and imaging research, the development of MRI-defined BML research as a distinct field has not been comprehensively mapped. The literature spans rheumatology, radiology, orthopedics, imaging methodology, pain research, subchondral biology, and clinical trials. It also uses overlapping terminology, including BML, bone marrow edema, bone marrow edema-like lesion, and subchondral marrow abnormality. Narrative reviews can synthesize selected evidence but cannot readily show how the field has evolved, which references form its intellectual foundation, which collaborative groups have shaped its development, or how research themes have shifted over time.

Bibliometric and science-mapping approaches can address this gap by characterizing publication trends, collaboration structures, influential references, co-citation relationships, term evolution, and emerging research directions (11). Previous bibliometric studies have examined selected osteoarthritis-related topics, including regenerative treatments and oxidative stress (12, 13). MRI-defined BML research differs because it centers on a specific imaging phenotype that connects clinical symptoms, whole-joint structural change, subchondral biology, imaging methodology, and therapeutic investigation. Mapping this phenotype as a distinct field can reveal how MRI-defined BMLs have connected otherwise dispersed clinical, structural, mechanistic, and translational research streams over time. Such a field-specific analysis requires integration of the field's intellectual, social, temporal, and conceptual structures beyond conventional productivity and citation rankings.

This study aimed to construct an integrated knowledge map of MRI-defined BML research in knee osteoarthritis using a deduplicated corpus from Web of Science Core Collection, Scopus, and PubMed. We combined descriptive bibliometrics, collaboration-network analysis, reference co-citation analysis, harmonized keyword and MeSH mapping, temporal thematic analysis, methodologically evaluated term frequency–inverse document frequency (TF-IDF)-based document clustering, and dictionary-based bridge-theme mapping. The objectives were to characterize the field's publication landscape and collaborative structure, identify its intellectual foundations, examine temporal changes in research emphasis, organize the literature into interpretable text-derived domains, and clarify how MRI-defined BMLs have become connected with pain, structural progression, subchondral remodeling, and treatment-oriented research.

## 2 Materials and methods

### 2.1 Study design and data sources

This study was conducted as a multi-database bibliometric and text-mining analysis of MRI-defined BML research in knee osteoarthritis. The study-identification and selection process was summarized in a flow diagram. Web of Science Core Collection (WoSCC), Scopus, and PubMed were searched from database inception on July 15, 2026. Eligibility was restricted to publications available on or before the prespecified cutoff date of June 1, 2026.

The final search strategy was organized around three core concepts: knee osteoarthritis, MRI, and BMLs or related bone marrow edema-like abnormalities. Controlled vocabulary and free-text terms were adapted to the indexing and syntax of each database. Pain, structural progression, treatment, cartilage loss, joint-space narrowing, and subchondral bone were not required search components. The complete database-specific strategies, filters, search dates, and export fields are provided in Supplementary Table S1.

The searches initially identified 2,452 records, including 837 from WoSCC, 848 from Scopus, and 767 from PubMed. After application of the prespecified document-type and language filters where supported, 2,415 records were exported for integration, including 800 from WoSCC, 848 from Scopus, and 767 from PubMed. WoSCC records were exported as full records with cited references. Scopus records included citation information, abstracts, author and indexed keywords, affiliations, identifiers, and cited references when available. PubMed records included PMID, DOI, title, abstract, authors, affiliations, journal information, publication type, and MeSH terms. The study-selection process and database overlap are shown in Figure 1 and summarized in Supplementary Table S2.

**Figure 1:**
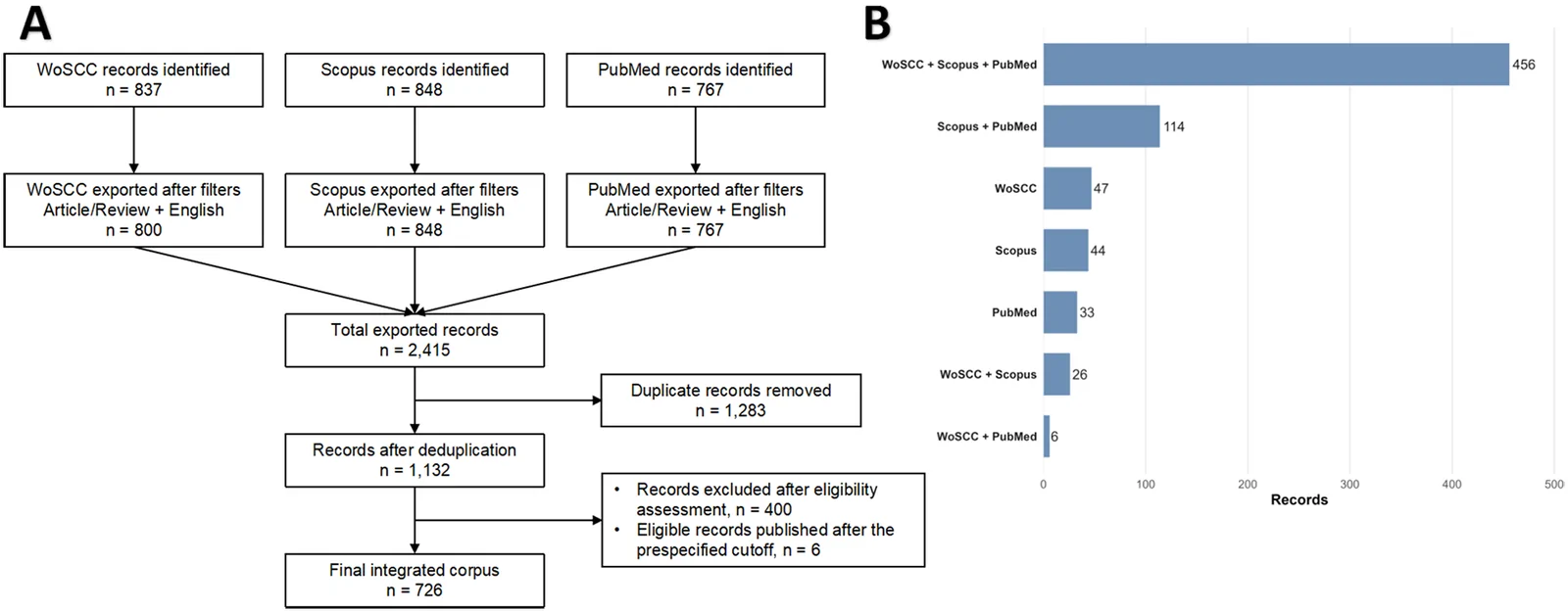
Study identification, selection, and database overlap. **(A)** Records identified from Web of Science Core Collection, Scopus, and PubMed were filtered, integrated, deduplicated, assessed against the eligibility criteria, and restricted to publications available by the prespecified cutoff date of June 1, 2026. The searches initially identified 2,452 records, 2,415 records were exported after database-level filters, and the final integrated corpus comprised 726 publications. **(B)** Distribution of the included publications across the three databases, including publications indexed in all three databases, pairs of databases, or a single database. WoSCC, Web of Science Core Collection.

### 2.2 Data integration and deduplication

All exported records were imported into R and harmonized into a common bibliographic structure. Core fields included title, publication year, journal, DOI, PMID, abstract, authors, affiliations, countries or regions, author keywords, indexed keywords or Keywords Plus, MeSH terms, cited references, and citation counts.

Duplicate records were identified hierarchically. Exact DOI matching was performed first after normalization of DOI strings, followed by exact PMID matching and exact matching of normalized titles. Remaining candidate pairs were assessed using Jaro-Winkler title similarity. The predefined similarity threshold was 0.95. Candidate pairs were retained only when the normalized title contained at least 30 characters, the first-author surname was compatible, and the publication-year difference was no greater than one year. Accepted exact and fuzzy links were consolidated into canonical records, and ambiguous candidate pairs were reviewed before merging. Database-source membership was retained for each integrated publication. The matching hierarchy and parameters are provided in Supplementary Table S4.

### 2.3 Eligibility assessment

Publications were eligible when they addressed knee osteoarthritis, an osteoarthritis-relevant experimental model, or a knee cohort with explicit relevance to osteoarthritis development or progression; involved MRI-defined or imaging-relevant BMLs, bone marrow edema-like lesions, subchondral marrow lesions, or an equivalent explicit construct; and treated BMLs as a substantive exposure, outcome, predictor, imaging phenotype, treatment target, or review topic. Eligible research directions included pain, symptoms, function, structural progression, cartilage loss, subchondral remodeling, treatment or intervention, imaging phenotyping, and related pathophysiology. Original research articles and reviews were eligible.

Publications were excluded when they concerned non-knee sites without separable knee data; lacked a knee osteoarthritis or osteoarthritis-relevant context; mentioned BMLs only in background text, indexing terms, adjustment models, or inseparable composite scores; or addressed acute traumatic, postoperative, or other marrow abnormalities without an interpretable osteoarthritis-related component.

Surgical involvement was not itself an exclusion criterion. Studies of arthroplasty, subchondroplasty, osteotomy, post-traumatic osteoarthritis, or other procedures were eligible when MRI-defined BMLs contributed to an osteoarthritis-related question concerning symptoms, structural phenotype, progression, or treatment response. Studies limited to isolated acute injury, reconstruction-related edema, or postoperative marrow changes without osteoarthritis relevance were excluded. Mixed populations were retained only when the osteoarthritis population was dominant or osteoarthritis-specific findings were reported separately. Full texts were examined when titles, abstracts, keywords, and indexing information were insufficient for a definitive decision. Eligibility criteria and representative boundary examples are provided in Supplementary Table S3.

### 2.4 Term harmonization and metadata preparation

Lexical variants and closely synonymous expressions in author keywords, indexed terms, and MeSH fields were harmonized before co-occurrence and temporal analyses. Normalization included lowercase conversion, punctuation and whitespace standardization, abbreviation expansion, spelling harmonization, and consolidation of singular, plural, and equivalent expressions. Examples included knee OA and knee osteoarthritis, MRI and magnetic resonance imaging, bone marrow lesion and BML, and bone marrow edema and bone marrow oedema.

Broader concept families, such as pain-related outcomes, structural progression, cartilage abnormalities, subchondral remodeling, and treatment-related concepts, were used only for descriptive thematic summaries and dictionary-based mapping. These concept families were not treated as interchangeable lexical synonyms and were not collapsed into single TF-IDF tokens. Generic demographic, indexing, and reporting terms with limited thematic specificity were removed from thematic visualization. Corpus-defining terms were retained in descriptive keyword and MeSH maps because their frequencies and relationships remained substantively relevant, whereas the most ubiquitous search-defining anchors were removed from the free-text TF-IDF vocabulary to prevent near-universal terms from dominating document separation. Lexical harmonization examples, descriptive concept families, removed generic terms, and analysis-specific handling of topic anchors are provided in Supplementary Table S5.

### 2.5 Descriptive bibliometric analysis

Annual and cumulative publication counts were calculated for the final corpus. Journals, countries or regions, institutions, and authors were ranked using full counting at the publication level. A publication could therefore contribute to more than one country, institution, or author count.

Institutional and author names were harmonized to reduce fragmentation caused by abbreviations, spelling variants, initials, and parent-system naming. Metadata completeness was evaluated for DOI, PMID, abstract, author keywords, indexed keywords or Keywords Plus, MeSH terms, and cited references. Metadata completeness and extended entity rankings are reported in Supplementary Tables S10 and S11. The bibliometric landscape is shown in Figure 2.

**Figure 2:**
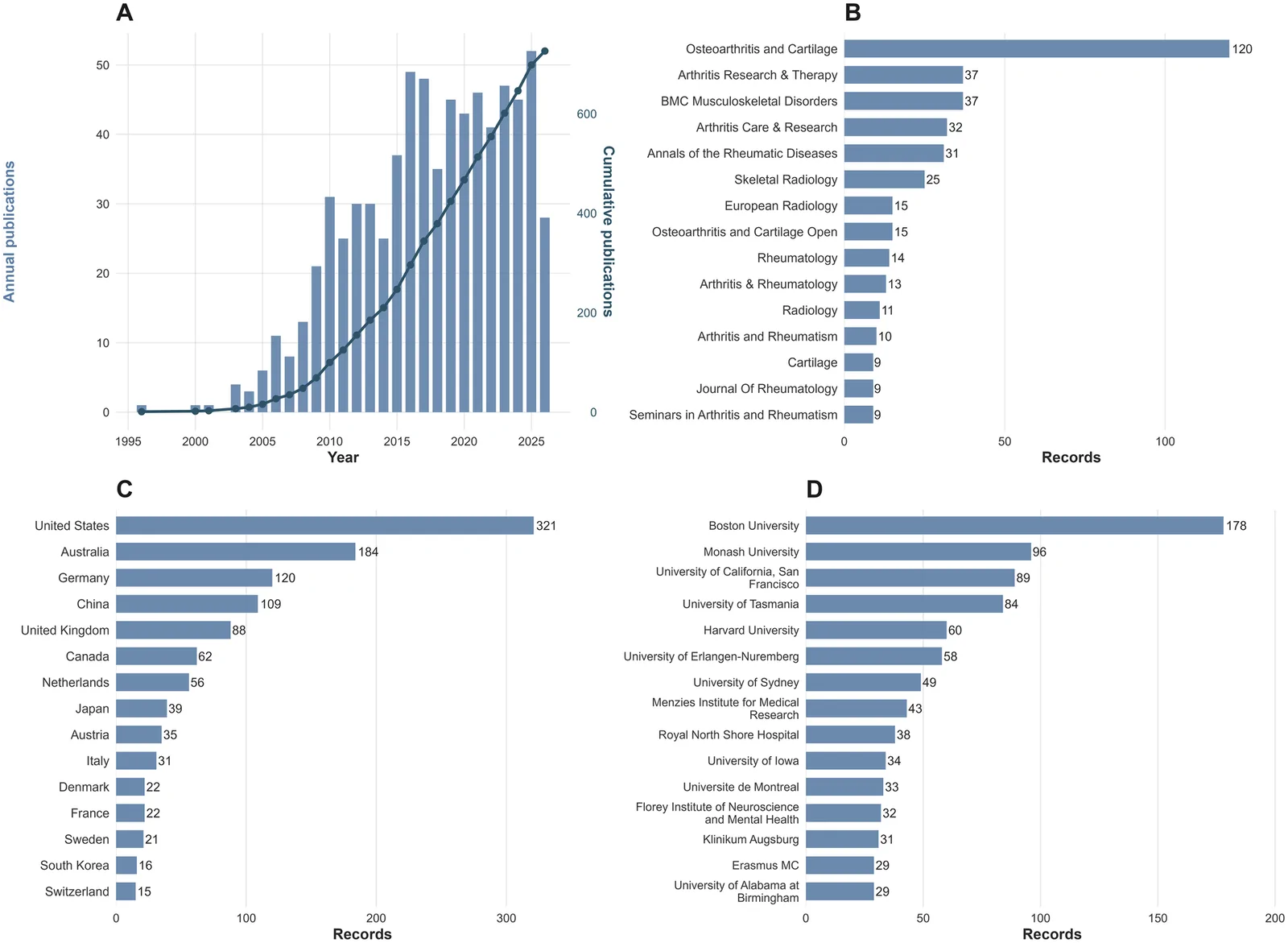
Bibliometric landscape of MRI-defined bone marrow lesion research in knee osteoarthritis. **(A)** Annual and cumulative publication output from 1996 to 2026. The 2026 count includes publications available by June 1 and represents an incomplete publication year. **(B)** Leading journals by number of included publications. **(C)** Leading contributing countries or regions. **(D)** Leading contributing institutions after affiliation-name harmonization. BML, bone marrow lesion; MRI, magnetic resonance imaging.

### 2.6 Collaboration-network analysis

Collaboration networks were constructed at the country or region, institution, and author levels. Nodes represented contributing entities, and undirected weighted edges represented their co-occurrence within the same publication. Edge weight equaled the number of shared publications.

At the country level, each publication was additionally classified as a single-country or multi-country publication for every participating country or region. The multi-country publication ratio was calculated as the number of multi-country publications divided by the total number of publications involving that country or region. Network characteristics and international-collaboration metrics are provided in Supplementary Table S12. Collaboration networks are shown in Figure 3.

**Figure 3:**
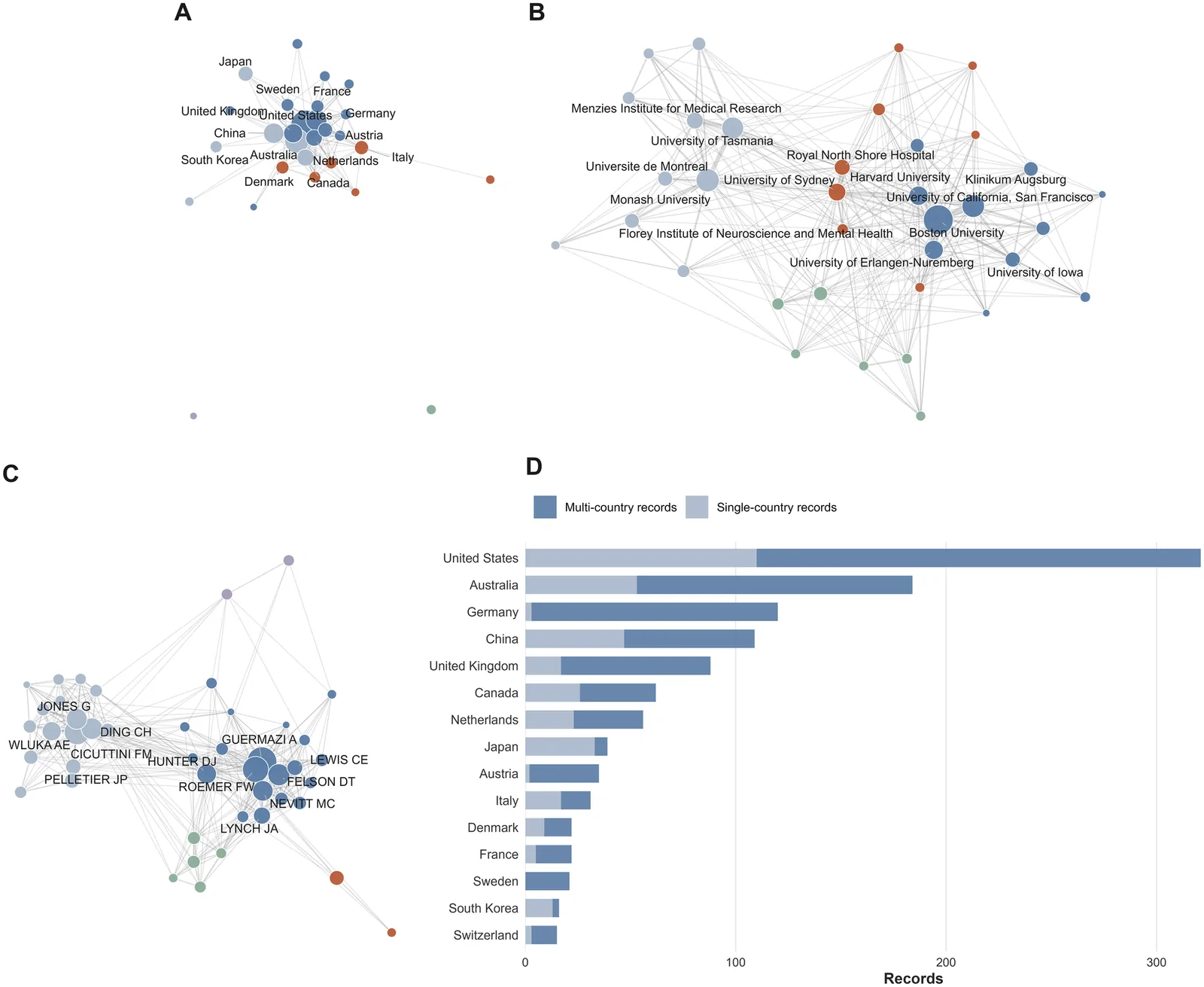
Collaboration structure of MRI-defined bone marrow lesion research in knee osteoarthritis. **(A)** Country- or region-level collaboration network. **(B)** Institution-level collaboration network. **(C)** Author-level collaboration network. Node size represents publication frequency, and edge width represents collaboration frequency. **(D)** Single-country and multi-country publication counts among leading contributing countries or regions. BML, bone marrow lesion; MCP, multi-country publication; SCP, single-country publication.

### 2.7 Knowledge-base and co-citation analysis

Cited-reference strings were parsed and standardized using the available first author, publication year, source title, DOI, and raw reference information. Local citation frequency was defined as the number of publications in the final corpus citing a given reference. References cited by at least five corpus publications were retained in the extended knowledge-base output.

Reference co-citation relationships were generated when two cited references appeared in the same corpus publication. Edge weight represented co-citation frequency. The final visualization retained 52 valid and highly connected references with identifiable publication year and source information after exclusion of three incomplete author-only reference strings. Normalized betweenness centrality was used to identify references occupying connecting positions within the network.

Annual local-citation profiles were evaluated to identify periods of concentrated influence. When a reference had more than one high-intensity period, the strongest period was retained in the principal summary. This analysis provided a corpus-specific description of temporally concentrated citation influence and was not presented as an exact replication of a proprietary citation-burst algorithm. Knowledge-base results are reported in Supplementary Table S13 and Figure 4.

**Figure 4:**
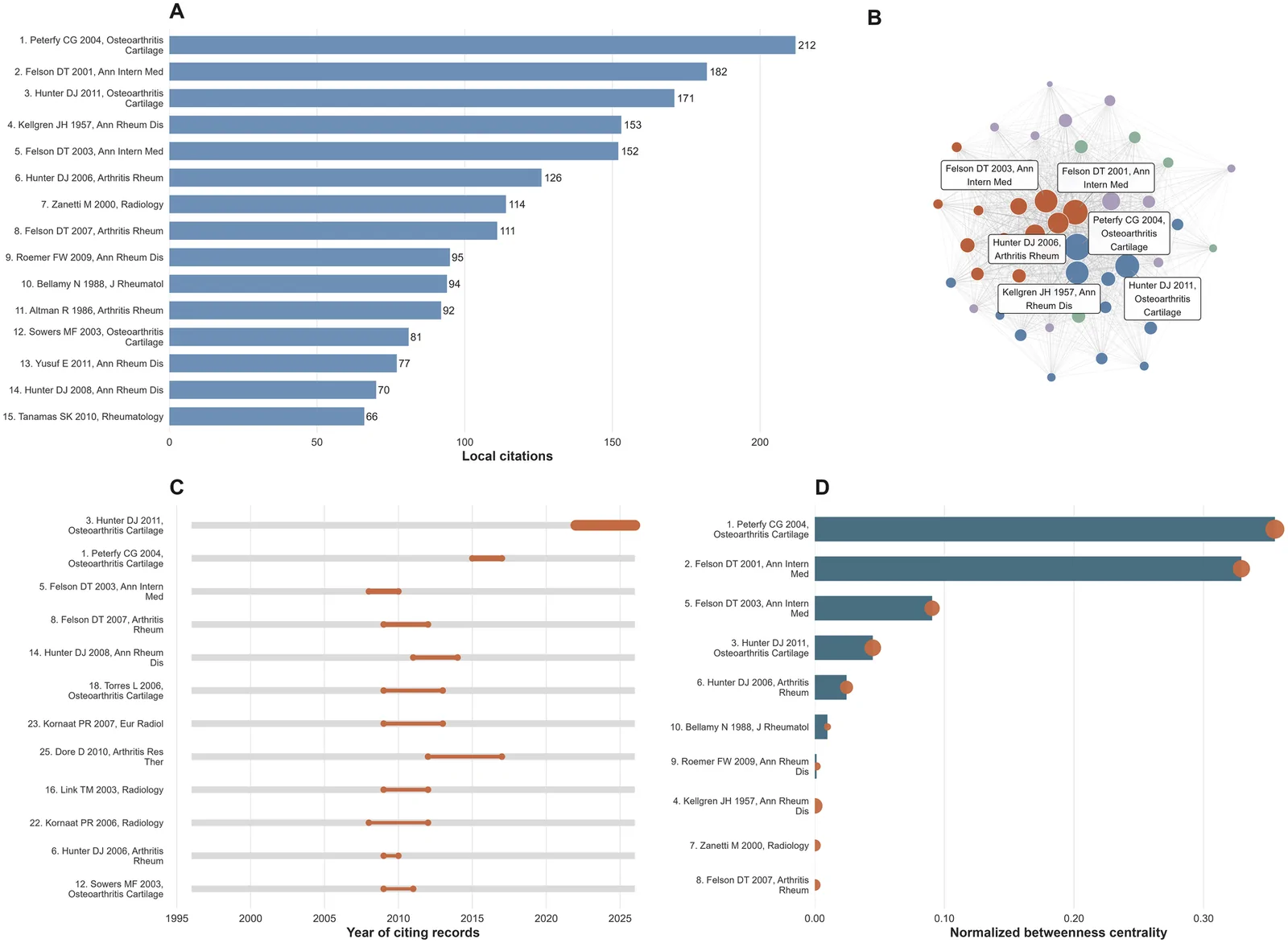
Intellectual base and reference co-citation structure. **(A)** Leading locally cited references within the final corpus. **(B)** Reference co-citation network of 52 valid and highly connected references; node size represents local citation frequency, and edge width represents co-citation frequency. **(C)** References with high-intensity local-citation periods. **(D)** Turning-point references ranked by normalized betweenness centrality within the co-citation network.

### 2.8 Keyword, MeSH, and temporal thematic analysis

Harmonized author keywords, indexed terms, and MeSH terms were analyzed using document frequency and record-level co-occurrence. Node size represented document frequency, and edge weight represented the number of publications in which two terms occurred together.

Temporal evolution was examined across four phase-based periods: 1996–2008, 2009–2014, 2015–2020, and 2021–2026. These periods were selected to reflect recognizable shifts in the dominant research emphasis of the field rather than statistically estimated change points. They represented the foundational phase of MRI characterization and early pain and progression studies; expansion of whole-joint MRI cohorts and semi-quantitative scoring; methodological, longitudinal, and translational maturation; and the contemporary phase of mechanistic, quantitative, and intervention-oriented research. The four periods contained 48, 162, 257, and 259 publications, respectively.

Selected harmonized terms were counted within each period. A predefined dictionary was also used to estimate the period-specific prevalence of six non-mutually exclusive temporal themes: pain and symptoms; structural progression; subchondral remodeling; synovitis and effusion; treatment and translation; and meniscus and biomechanics. The rationale for the period definitions and the complete period-level results are provided in Supplementary Table S6. Temporal patterns are shown in Figure 5.

**Figure 5:**
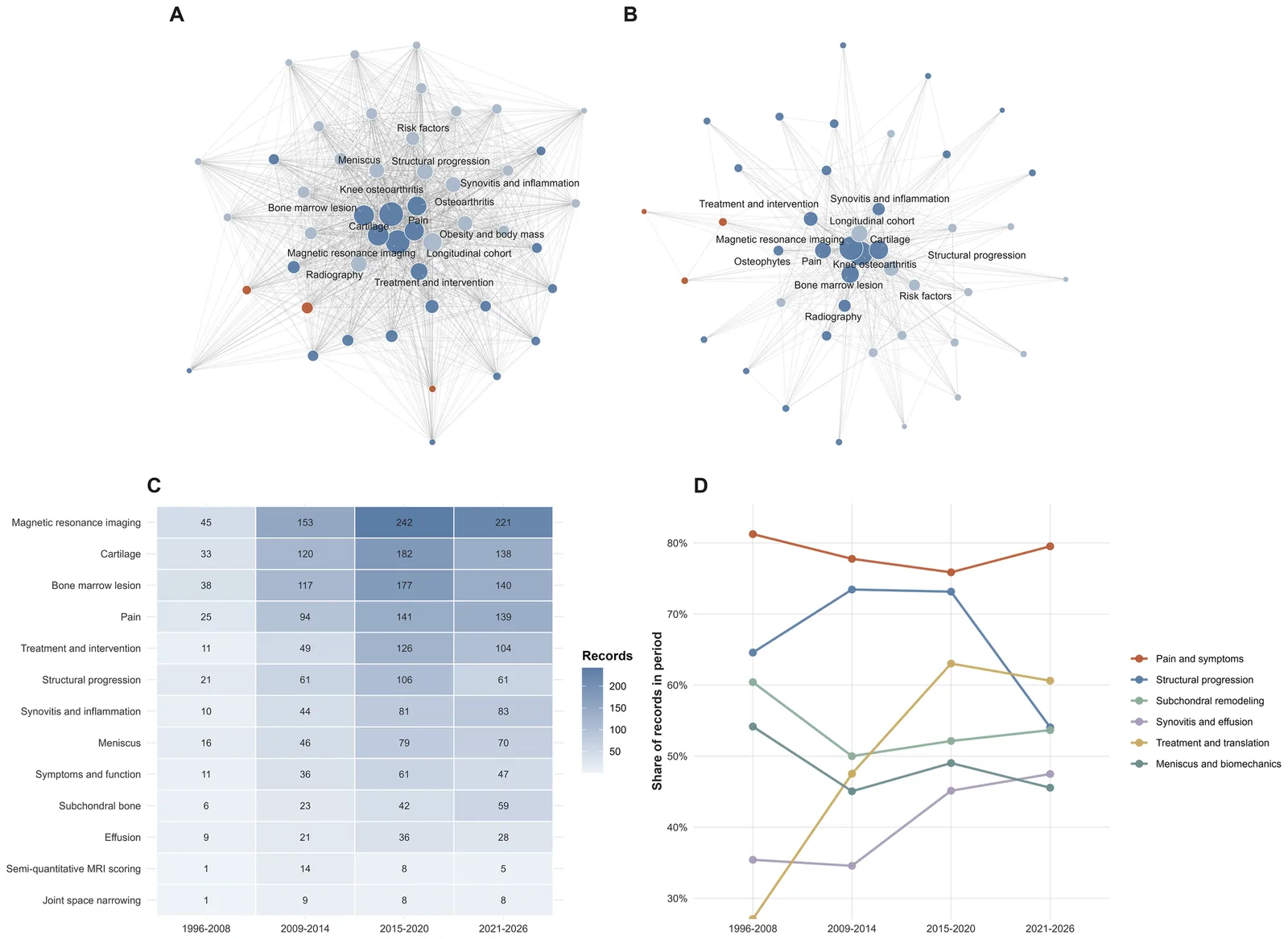
Keyword, MeSH, and temporal thematic evolution of MRI-defined bone marrow lesion research. **(A)** Co-occurrence network of harmonized author keywords and indexed terms. **(B)** Co-occurrence network of harmonized MeSH terms. **(C)** Distribution of selected terms across four periods: 1996−2008, 2009−2014, 2015−2020, and 2021−2026. **(D)** Period-specific prevalence of six predefined, non-mutually exclusive temporal themes. Node size in the network panels represents document frequency, and edge width represents term co-occurrence frequency. BML, bone marrow lesion; MeSH, Medical Subject Headings; MRI, magnetic resonance imaging.

### 2.9 TF-IDF-based document clustering

Document-level text was formed by concatenating titles, abstracts, author keywords, indexed keywords, and MeSH-derived terms when available. Text was converted to lowercase, punctuation and repeated whitespace were normalized, and alphanumeric single-word tokens containing at least three characters were extracted. Standard stopwords, generic biomedical reporting terms, and highly prevalent corpus-defining anchors were excluded from the clustering vocabulary.

Stemming and lemmatization were not applied because preservation of readable biomedical terminology was considered important for interpretation. Automatic statistical phrase detection was not used. Clinically meaningful multiword concepts were represented through predefined harmonization of structured terms and transparent dictionary expressions. Complete preprocessing decisions are provided in Supplementary Table S7.

Tokens were required to occur in at least four publications and in no more than 65% of the corpus. Eligible tokens were ordered by document frequency. Term frequency-inverse document frequency values were calculated, zero-variance features were removed, and the resulting document-feature matrix was standardized. The 900 highest-frequency eligible tokens were used in the primary analysis.

K-means clustering was performed with 80 random starts and a maximum of 200 iterations. Candidate solutions from k = 2 to k = 10 were evaluated using total within-cluster sum of squares, the ratio of between-cluster to total sum of squares, mean silhouette width, gap statistics, and repeated-subsample stability. Gap statistics were estimated using 30 reference datasets and the first 100 principal components, which retained 46.9% of the total variance for computational tractability.

For stability assessment, 20 random subsamples containing 80% of the publications were generated for each value of k. Each subsample was reclustered, all publications were assigned to the nearest subsample centroid, and agreement with the full-corpus solution was quantified using the adjusted Rand index.

No single diagnostic identified a uniquely optimal cluster number. The six-cluster solution was retained as an interpretability-oriented intermediate-granularity representation because it provided clinically coherent research domains with moderate resampling stability. The domains were treated as exploratory and overlapping research structures, not as sharply separated natural classes.

Vocabulary-size sensitivity was evaluated using the 600, 900, and 1,200 highest-frequency eligible tokens at k = 6. The 900-token vocabulary covered 79.0% of cumulative document-frequency mass and 81.1% of token occurrences. The corresponding values were 69.2% and 72.2% for 600 tokens and 85.6% and 87.1% for 1,200 tokens. Cluster-number diagnostics, resampling stability, and vocabulary sensitivity results are reported in Supplementary Table S8 and Supplementary Figure S2.

Principal component analysis was used only for visualization. The first and second principal components explained 1.65% and 1.24% of the total variance, respectively. Cluster assignment was performed in the complete standardized 900-token TF-IDF feature space and not in the two-dimensional principal-component projection.

### 2.10 Semantic labeling and bridge-theme mapping

Research-domain labels were assigned after joint examination of the leading discriminating TF-IDF terms, publications nearest each cluster centroid, and the distribution of bridge themes within each cluster. The labels were descriptive interpretations of dominant publication patterns rather than externally validated disease classifications.

A predefined dictionary was used to identify eight bridge themes: imaging-defined BMLs; pain and symptoms; structural progression; treatment and translation; subchondral remodeling; meniscus and biomechanics; synovitis, effusion, and inflammation; and neurovascular mechanisms. Dictionary construction was informed by representative publications, harmonized terminology, high-discrimination text terms, and established osteoarthritis concepts.

A publication was assigned to a theme when one or more predefined expressions were detected in its combined title, abstract, keyword, indexed-term, or MeSH text. Multiple theme assignments were permitted. The complete dictionary and matching expressions are provided in Supplementary Table S9. Research-domain and domain-theme results are reported in Supplementary Table S14 and Figure 6.

**Figure 6:**
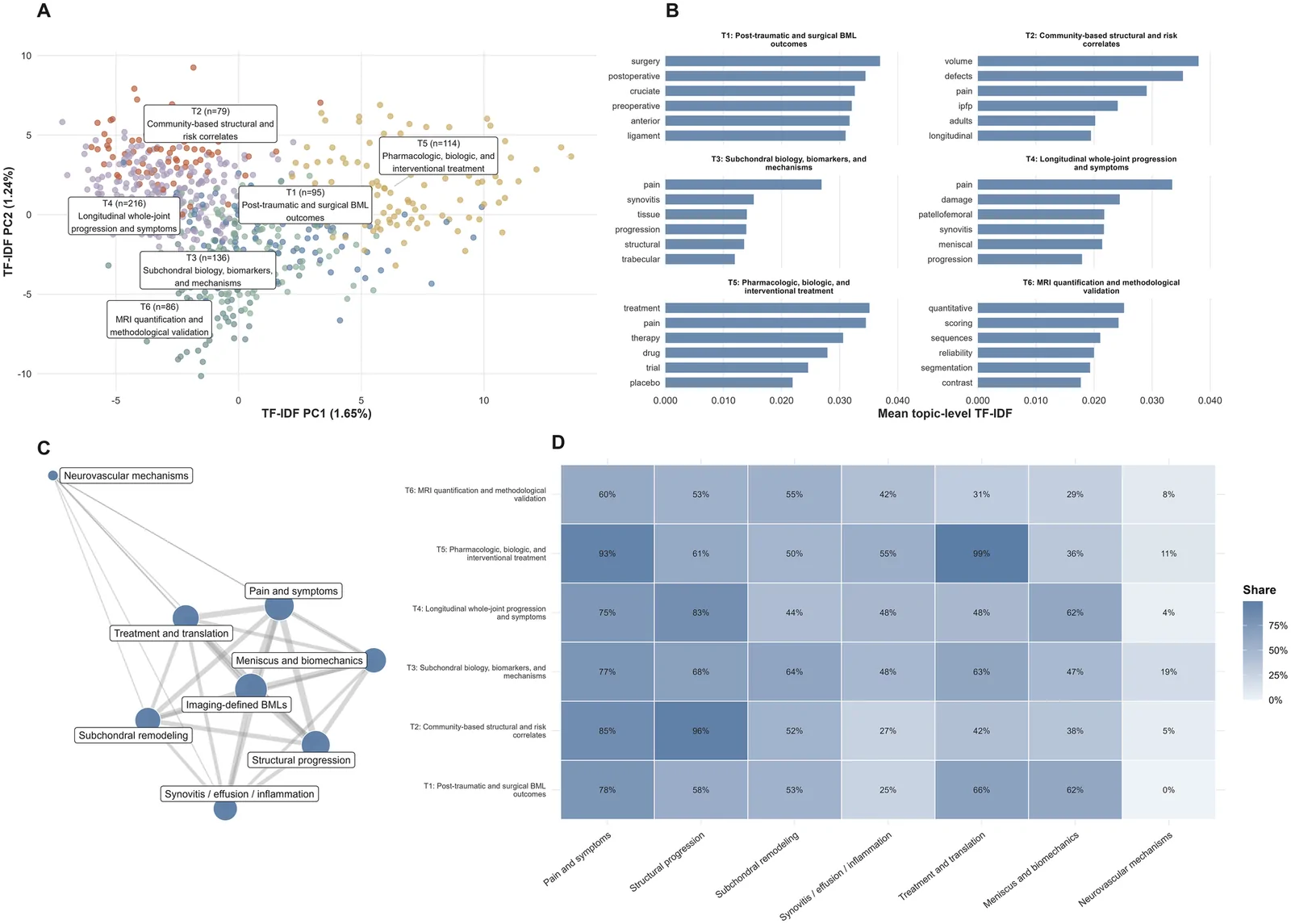
Text-derived research domains and bridge-theme relationships. **(A)** Principal-component visualization of the six exploratory research domains identified from the complete standardized 900-token TF-IDF feature space. The first two components explained 1.65% and 1.24% of the total variance, respectively, and were used only for visualization. **(B)** Leading substantive discriminating terms for each research domain. Generic connective or time words were omitted from the panel display only and replaced by the next-ranked substantive terms; TF-IDF calculations and cluster assignments were unchanged. **(C)** Network connecting the six research domains with predefined clinical, structural, mechanistic, and translational bridge themes. **(D)** Prevalence of bridge themes within each research domain. The universal Imaging-defined BMLs theme, which was present in all 726 publications, was omitted from panel D because it was non-discriminating across research domains; it remains included in panel C and Supplementary Tables S14C–D. Full semantic domain labels are used throughout the figure and Supplementary Table S14. Theme assignments were non-mutually exclusive. IPFP, infrapatellar fat pad; TF-IDF, term frequency-inverse document frequency.

### 2.11 Sensitivity analyses

The 2026 publication count was incomplete because eligibility was restricted to publications available by June 1, 2026. Temporal analyses were therefore repeated after exclusion of all 2026 publications. Annual publication trends, leading recent terms, and period-level temporal-theme prevalence were compared with the primary analysis.

Clustering robustness was additionally examined through the candidate k analyses, repeated 80% subsampling, and vocabulary-size sensitivity analyses described above.

### 2.12 Software

All analyses and figure generation were performed in R version 4.5.1. Principal packages included bibliometrix, tidyverse, dplyr, tidyr, purrr, tibble, readr, stringr, stringi, janitor, ggplot2, ggrepel, patchwork, scales, igraph, cluster, showtext, sysfonts, and showtextdb.

## 3 Results

### 3.1 Corpus construction and study selection

The searches initially identified 2,452 records, including 837 from WoSCC, 848 from Scopus, and 767 from PubMed. After the prespecified database-level filters were applied, 2,415 records were exported for integration, including 800 from WoSCC, 848 from Scopus, and 767 from PubMed. Sequential within-database and cross-database deduplication removed 1,283 records, leaving 1,132 unique candidate records for eligibility assessment.

Of the 1,132 unique records, 400 were excluded because they did not meet the disease-context, BML-role, anatomical-site, or publication-type criteria. Six additional content-eligible publications were excluded because they were published after the prespecified cutoff date. The final integrated corpus comprised 726 publications (Figure 1A; Supplementary Table S2).

Database overlap was substantial. Among the 726 included publications, 456 were represented in WoSCC, Scopus, and PubMed; 114 in Scopus and PubMed; 26 in WoSCC and Scopus; and 6 in WoSCC and PubMed. Among publications represented in only one database, 47 were unique to WoSCC, 44 to Scopus, and 33 to PubMed (Figure 1B).

### 3.2 Bibliometric landscape

The final corpus covered publications from 1996 through 2026. Annual output was limited during the earliest years, increased with the wider adoption of semi-quantitative whole-joint MRI assessment and longitudinal osteoarthritis cohorts, and expanded further after 2015. The 2026 count comprised publications available through the June 1 cutoff and therefore represented an incomplete publication year (Figure 2A).

Metadata coverage was high for the principal bibliographic fields. DOI information was available for 713 publications (98.2%), PMID for 678 (93.4%), abstracts for 724 (99.7%), author keywords for 541 (74.5%), indexed keywords or Keywords Plus for 680 (93.7%), MeSH terms for 517 (71.2%), and cited-reference strings for 715 (98.5%) (Supplementary Table S10).

Osteoarthritis and Cartilage was the leading journal, with 120 publications, corresponding to 16.5% of the corpus. It was followed by Arthritis Research & Therapy and BMC Musculoskeletal Disorders (*n* = 37 each), Arthritis Care & Research (*n* = 32), Annals of the Rheumatic Diseases (*n* = 31), Skeletal Radiology (*n* = 25), European Radiology and Osteoarthritis and Cartilage Open (*n* = 15 each), Rheumatology (*n* = 14), and Arthritis & Rheumatology (*n* = 13) (Figure 2B; Supplementary Table S11).

The United States contributed to 321 publications, followed by Australia (*n* = 184), Germany (*n* = 120), China (*n* = 109), the United Kingdom (*n* = 88), Canada (*n* = 62), the Netherlands (*n* = 56), Japan (*n* = 39), Austria (*n* = 35), and Italy (*n* = 31) (Figure 2C).

The leading harmonized institutions were Boston University (*n* = 178), Monash University (*n* = 96), the University of California, San Francisco (*n* = 89), the University of Tasmania (*n* = 84), Harvard University (*n* = 60), the University of Erlangen-Nuremberg (*n* = 58), the University of Sydney (*n* = 49), the Menzies Institute for Medical Research (*n* = 43), Royal North Shore Hospital (*n* = 38), and the University of Iowa (*n* = 34) (Figure 2D; Supplementary Table S11).

### 3.3 Collaboration structure

The complete country or region collaboration network contained 32 nodes and 150 edges. The institution network contained 1,283 nodes and 7,785 edges, and the author network contained 2,564 nodes and 14,879 edges (Supplementary Table S12).

The country-level network was centered on the United States, Australia, Germany, China, and the United Kingdom (Figure 3A). Boston University, Monash University, the University of California, San Francisco, the University of Tasmania, Harvard University, and the University of Erlangen-Nuremberg occupied prominent positions in the institutional network (Figure 3B).

After author-name harmonization, the leading contributors were A. Guermazi (*n* = 146), F. W. Roemer (*n* = 106), F. M. Cicuttini (*n* = 99), D. T. Felson (*n* = 69), C. H. Ding (*n* = 66), G. Jones (*n* = 62), M. C. Nevitt (*n* = 59), D. J. Hunter (*n* = 53), A. E. Wluka (*n* = 50), and J. A. Lynch (*n* = 38) (Figure 3C; Supplementary Table S11).

International collaboration varied across countries. Multi-country publications accounted for 97.5% of Germany's publications, 94.3% of Austria's, 80.7% of the United Kingdom's, 71.2% of Australia's, and 65.7% of the United States’ publications. Japan had a lower multi-country publication ratio of 15.4% (Figure 3D; Supplementary Table S12).

### 3.4 Knowledge base and co-citation structure

The 726 publications contained 31,319 parsed cited-reference occurrences and 16,708 unique cited references. A total of 906 references received at least five local citations. Following exclusion of three incomplete author-only reference strings lacking identifiable publication year and source information, the final co-citation visualization contained 52 references and 1,158 edges (Figure 4; Supplementary Table S13).

The most locally cited reference was Peterfy et al. 2004 in Osteoarthritis and Cartilage (*n* = 212), followed by Felson et al. 2001 in Annals of Internal Medicine (*n* = 182), Hunter et al. 2011 in Osteoarthritis and Cartilage (*n* = 171), Kellgren and Lawrence 1957 in Annals of the Rheumatic Diseases (*n* = 153), Felson et al. 2003 in Annals of Internal Medicine (*n* = 152), and Hunter et al. 2006 in Arthritis & Rheumatism (*n* = 126) (Figure 4A). Zanetti et al. 2000, Felson et al. 2007, and Roemer et al. 2009 were also among the most frequently cited references.

The co-citation network showed dense relationships among references addressing whole-joint MRI scoring, BML-associated pain, cartilage loss, structural progression, and standardized osteoarthritis assessment (Figure 4B). Peterfy et al. 2004 had the highest normalized betweenness centrality (0.355), followed by Felson et al. 2001 (0.329), Felson et al. 2003 (0.091), Hunter et al. 2011 (0.045), and Hunter et al. 2006 (0.025), indicating their connecting positions within the knowledge base (Figure 4D).

Analysis of high-intensity local-citation periods identified both historical and continuing influence. Hunter et al. 2011 had the strongest period from 2022 through 2026, with a local-citation-period strength of 49.4. Peterfy et al. 2004 showed a strong period from 2015 through 2017, whereas Felson et al. 2003, Felson et al. 2007, Hunter et al. 2008, Torres et al. 2006, Kornaat et al. 2007, and Link et al. 2003 showed concentrated influence during earlier phases of the field (Figure 4C; Supplementary Table S13).

### 3.5 Keyword, MeSH, and temporal thematic evolution

Keyword and indexed-term co-occurrence analysis showed a dense central structure organized around knee osteoarthritis, MRI, cartilage, BMLs, pain, structural progression, longitudinal cohorts, radiography, risk factors, synovitis and inflammation, meniscal abnormalities, and treatment or intervention (Figure 5A). The MeSH network showed a comparable whole-joint structure, with knee osteoarthritis, MRI, cartilage, BMLs, pain, osteophytes, radiography, synovitis and inflammation, and longitudinal-study concepts among the principal connected terms (Figure 5B).

The four temporal periods contained 48 publications in 1996–2008, 162 in 2009–2014, 257 in 2015–2020, and 259 in 2021–2026. MRI-related terms occurred in 45, 153, 242, and 221 publications across the four periods, respectively. The corresponding period counts were 33, 120, 182, and 138 for cartilage; 38, 117, 177, and 140 for BML; 25, 94, 141, and 139 for pain; 11, 49, 126, and 104 for treatment and intervention; 21, 61, 106, and 61 for structural progression; and 6, 23, 42, and 59 for subchondral bone (Figure 5C; Supplementary Table S6).

Pain and symptoms remained the most prevalent temporal theme throughout the study period, occurring in 81.3%, 77.8%, 75.9%, and 79.5% of publications across the four periods. Structural progression was present in 64.6%, 73.5%, 73.2%, and 54.1%, respectively. Subchondral remodeling occurred in 60.4%, 50.0%, 52.1%, and 53.7%; synovitis and effusion in 35.4%, 34.6%, 45.1%, and 47.5%; treatment and translation in 27.1%, 47.5%, 63.0%, and 60.6%; and meniscus and biomechanics in 54.2%, 45.1%, 49.0%, and 45.6% (Figure 5D; Supplementary Table S6).

The lower prevalence of the structural-progression theme during 2021–2026 occurred alongside sustained treatment-oriented research, increasing representation of synovitis and effusion, and stable subchondral-remodeling prevalence. This pattern was consistent with diversification of the field while progression-oriented research remained an important component.

### 3.6 Text-derived research domains

TF-IDF-based clustering assigned all 726 publications to six exploratory research domains (Figure 6A). The largest domain was longitudinal whole-joint progression and symptoms (*n* = 216), followed by subchondral biology, biomarkers, and mechanisms (*n* = 136); pharmacologic, biologic, and interventional treatment (*n* = 114); post-traumatic and surgical BML outcomes (*n* = 95); MRI quantification and methodological validation (*n* = 86); and community-based structural and risk correlates (*n* = 79).

The post-traumatic and surgical BML outcomes domain was characterized by terms including surgery, postoperative, cruciate, preoperative, ligament, and subchondroplasty. The community-based structural and risk-correlate domain included volume, defects, pain, infrapatellar fat pad, adults, and population-related terms. The subchondral biology, biomarkers, and mechanisms domain emphasized pain, synovitis, tissue, trabecular characteristics, structural progression, biomarkers, and mechanistic terminology.

The longitudinal whole-joint progression and symptoms domain was characterized by pain, damage, patellofemoral involvement, synovitis, meniscal pathology, and progression. The pharmacologic, biologic, and interventional treatment domain included treatment, therapy, drug, trial, placebo, and follow-up terminology. The MRI quantification and methodological validation domain emphasized quantitative imaging, scoring, sequences, reliability, segmentation, and contrast (Figure 6B; Supplementary Table S14).

### 3.7 Bridge-theme relationships

Bridge-theme mapping identified eight non-mutually exclusive themes and 28 domain-theme edges. Imaging-defined BML concepts were present in all 726 publications by design. Pain and symptoms occurred in 567 publications, structural progression in 518, treatment and translation in 425, subchondral remodeling in 377, meniscus and biomechanics in 353, synovitis/effusion/inflammation in 312, and neurovascular mechanisms in 57 (Figure 6C).

Each research domain was connected to several clinical, structural, and mechanistic themes (Figure 6D). Post-traumatic and surgical BML outcomes were linked particularly with pain and symptoms, treatment and translation, and meniscus and biomechanics. Community-based structural and risk correlates were linked predominantly with pain, structural progression, and whole-joint structural risk features.

Subchondral biology, biomarkers, and mechanisms showed comparatively strong connections with remodeling, inflammation, and neurovascular concepts. Longitudinal whole-joint progression and symptoms was strongly linked with pain, structural progression, meniscal pathology, and synovitis. The pharmacologic, biologic, and interventional treatment domain was dominated by treatment and translation but retained substantial pain and structural-progression components. MRI quantification and methodological validation connected imaging methods with pain, progression, and subchondral characterization.

These overlapping relationships support interpretation of the six clusters as descriptive research domains rather than mutually exclusive study categories.

### 3.8 Cluster-validation and vocabulary-sensitivity analyses

No individual cluster-number diagnostic identified a uniquely optimal solution (Supplementary Figure S2; Supplementary Table S8). The two-cluster solution had the highest mean silhouette width and the greatest repeated-subsample stability, whereas the gap statistic continued to increase through the upper evaluated boundary and selected k = 10. Elbow curvature identified k = 3, k = 4, and k = 8 as the strongest local candidates.

The six-cluster solution had a mean silhouette width of −0.0039 and a mean adjusted Rand index of 0.648 across 20 repeated 80% subsamples, with a minimum adjusted Rand index of 0.473. It was retained as an interpretable intermediate-granularity representation and not as a uniquely optimal or naturally separated partition.

Vocabulary sensitivity showed moderate agreement with the 900-token reference solution. The adjusted Rand index was 0.617 for the 600-token specification and 0.657 for the 1,200-token specification. The 900-token vocabulary covered 79.0% of cumulative document-frequency mass and 81.1% of token occurrences, intermediate between the 600-token specification, which covered 69.2% and 72.2%, and the 1,200-token specification, which covered 85.6% and 87.1%.

The first two principal components explained 1.65% and 1.24% of the total TF-IDF variance, respectively. Their combined contribution of 2.89% indicates that Figure 6A provides a two-dimensional visualization of the full-space clustering and does not represent the complete document-separation structure.

### 3.9 Partial-year sensitivity analysis

Twenty-eight publications were dated 2026, leaving 698 publications when the incomplete year was excluded. Across complete publication years from 1996 through 2025, annual publication output remained strongly correlated with publication year (Spearman *ρ*=0.946), compared with *ρ*=0.907 when the partial 2026 year was included. The corresponding linear slopes were 2.07 and 1.90 publications per year, respectively.

Exclusion of 2026 retained 29 of the 30 leading recent terms, corresponding to 96.7% retention. The largest absolute difference in recent temporal-theme prevalence was 2.22 percentage points. These findings indicate that the principal temporal and thematic results were not driven by inclusion of the partial 2026 publication year (Supplementary Figure S1; Supplementary Table S6).

## 4 Discussion

This study maps 726 publications on MRI-defined BMLs in knee osteoarthritis and provides three principal findings. First, the field is internationally collaborative but remains concentrated around several established osteoarthritis imaging groups and large longitudinal cohorts. Second, its intellectual base is anchored by standardized whole-joint MRI assessment and foundational studies linking BMLs with pain and structural progression. Third, contemporary research extends beyond lesion detection to subchondral biology, biomechanics, inflammation, quantitative imaging, and treatment-response phenotyping. The six text-derived domains provide an interpretive organization of these overlapping research directions, with each domain representing a dominant textual pattern within the literature. Together, they provide a field-level representation of how major lines of inquiry are distributed across this heterogeneous literature.

### 4.1 Research landscape and collaborative organization

The leading journals, institutions, and investigators show that MRI-defined BML research has developed across osteoarthritis, rheumatology, radiology, musculoskeletal imaging, orthopedics, and clinical-outcomes research. This multidisciplinary structure reflects the methodological requirements of BML studies, which commonly depend on standardized MRI acquisition, reproducible lesion definitions, trained image readers, longitudinal follow-up, and sufficiently large cohorts to examine symptom and structural outcomes.

The concentration of output among several established osteoarthritis imaging groups reflects the importance of large longitudinal cohorts and standardized whole-joint MRI assessment. Instruments such as WORMS and MOAKS provided a common framework for evaluating BMLs together with cartilage damage, meniscal pathology, synovitis, effusion, and other joint abnormalities (9, 10). International collaboration has supported methodological consistency and long-term cohort development, although broader geographical participation and closer integration of imaging, biomechanics, molecular biology, pain research, and clinical trials may improve external validity and facilitate multimodal BML phenotyping.

### 4.2 Intellectual foundations of measurement, pain, and progression

The co-citation structure indicates that the field matured through the convergence of three questions: how BMLs should be measured, whether they are clinically meaningful, and whether they are associated with subsequent structural deterioration. Standardized MRI scoring enabled comparable assessment across studies (9, 10), while foundational clinical studies established associations between BMLs, knee pain, and osteoarthritis progression (6, 7).

The persistence of pain-related terms across all four periods is consistent with the continuing clinical importance of BMLs. Nevertheless, the relationship between BMLs and pain is unlikely to be uniform. It may depend on lesion size, location, temporal change, adjacent cartilage and meniscal abnormalities, inflammatory features, local loading, and individual pain-processing mechanisms. BMLs should therefore not be interpreted as a direct or sufficient surrogate for pain severity.

Structural-progression research provided a second major foundation. Recent imaging reviews and longitudinal analyses continue to examine relationships between BML development or enlargement and subsequent osteoarthritis progression (14–17). Lesion trajectory may be more informative than baseline presence alone because incident, enlarging, persistent, resolving, and fluctuating lesions may represent different mechanical and biological states.

### 4.3 From an imaging abnormality to a whole-joint interpretive marker

The keyword, MeSH, and temporal analyses showed sustained emphasis on MRI, cartilage, BMLs, pain, and structural progression, accompanied by increasing representation of treatment, subchondral, inflammatory, and quantitative-imaging themes. This pattern reflects a transition from identifying whether a lesion is present to interpreting its meaning within the wider osteoarthritic joint.

Synovitis may partly mediate the association between BMLs and knee pain (18), while quantitative measures of effusion-synovitis and Hoffa-synovitis have been associated with pain and structural progression (19). Meniscal extrusion and related biomechanical abnormalities provide another contextual pathway because altered load distribution can affect cartilage and subchondral bone simultaneously (20). These observations support interpretation of BMLs together with adjacent tissue abnormalities.

A transient lesion in a relatively preserved compartment may have different implications from an enlarging lesion accompanied by cartilage denudation, meniscal extrusion, synovitis, malalignment, and sustained mechanical overload. Future clinical studies should therefore report lesion location, compartment, size, persistence, temporal change, and relationships with adjacent joint pathology. Such characterization is likely to be more informative than a binary present-or-absent classification.

### 4.4 Interpretation of the text-derived research domains

The largest domain, longitudinal whole-joint progression and symptoms, reflects the central role of cohort studies examining BMLs together with pain, cartilage damage, meniscal abnormalities, synovitis, and radiographic progression. This domain connects early symptom and progression research with contemporary whole-joint phenotyping and remains central to the clinical interpretation of BMLs.

The subchondral biology, biomarkers, and mechanisms domain represents increasing efforts to understand what the MRI signal reflects at tissue and molecular levels. Histopathological and mechanistic studies indicate that BMLs may contain combinations of trabecular abnormalities, fibrosis, necrosis, vascular alteration, microdamage, remodeling, immune-cell changes, and bone-cartilage interactions (8, 21–23). This biological heterogeneity may help explain why lesions with similar MRI appearances differ in symptoms, prognosis, and treatment response.

The pharmacologic, biologic, and interventional treatment domain shows that BMLs are increasingly used as eligibility features, imaging outcomes, and potential indicators of target engagement. Early randomized evidence suggested that zoledronic acid might reduce pain and BML size in selected patients (24), whereas a later randomized trial did not establish a corresponding disease-modifying effect on tibiofemoral cartilage volume (25). Studies of strontium ranelate and chondroitin sulphate have also reported changes in BML or cartilage outcomes in specific settings (26, 27). These findings support further evaluation of BMLs in treatment-response phenotyping but do not justify treating all BMLs as a uniform therapeutic target.

The MRI quantification and methodological validation domain reflects continued development of scoring systems, quantitative lesion measurement, segmentation, reliability assessment, sequence optimization, radiomics, and imaging applications in clinical trials (28, 29). This work remains important because observed associations depend on how lesions are defined, measured, and followed. Greater standardization of acquisition, lesion boundaries, quantitative-change thresholds, and reporting would improve comparability among cohorts and trials.

The community-based structural and risk-correlate domain extends BML research beyond specialized clinical cohorts by examining lesion volume, cartilage defects, body composition, infrapatellar fat pad characteristics, pain, and other population-level factors. This direction may help distinguish factors associated with lesion occurrence from those associated with persistence, symptom expression, or progression.

The post-traumatic and surgical BML outcomes domain requires careful contextual interpretation. Its presence does not indicate unrestricted inclusion of isolated traumatic or postoperative marrow edema. Eligible publications retained an osteoarthritis-related question, such as post-traumatic osteoarthritis development, BML-associated symptoms or structural change in an osteoarthritic joint, or treatment response in an osteoarthritis-relevant surgical population. This domain illustrates that BML research extends into perioperative and post-traumatic settings when marrow abnormalities remain connected to osteoarthritis phenotype, progression, or intervention response.

### 4.5 Mechanistic and translational implications

The bridge-theme map showed that pain and symptoms, structural progression, treatment and translation, subchondral remodeling, meniscus and biomechanics, and synovitis/effusion/inflammation were distributed across multiple research domains. This overlap supports the view that MRI-defined BMLs are shared imaging manifestations positioned at the intersection of several disease processes. More broadly, the cross-domain distribution of these themes indicates that the field is organized through interconnected clinical, structural, mechanistic, and translational lines of inquiry rather than isolated lesion-outcome relationships. This pattern supports longitudinal and multimodal study designs that examine BMLs within the evolving whole-joint and patient context.

Neurovascular terminology was less frequent than the major pain, progression, and treatment themes but identified a focused mechanistic direction. Neurovascular invasion at the osteochondral junction and peripheral neuronal sensitization provide plausible pathways linking subchondral change with pain (30, 31). Because this theme involved a comparatively small proportion of the corpus, it should be regarded as an emerging direction and not as a dominant organizing feature of BML research.

A productive translational direction is to move from generic BML identification toward longitudinal phenotyping. Mechanically driven lesions, inflammation-associated lesions, remodeling-dominant lesions, treatment-responsive lesions, and lesions associated with persistent pain mechanisms may overlap on conventional MRI but differ in natural history and therapeutic relevance. Combining quantitative MRI with biomechanical measures, synovial and subchondral biomarkers, pain phenotyping, and repeated lesion assessment may help resolve this heterogeneity.

Artificial intelligence may accelerate this transition by supporting more scalable and reproducible MRI-based phenotyping. Radiomics can extract quantitative characteristics from BMLs, subchondral bone, and other load-bearing joint tissues (28, 32, 33), while deep-learning methods may support automated BML segmentation, quantitative volume measurement, and longitudinal lesion assessment (17, 34). These approaches may also integrate imaging features with cartilage, meniscal, synovial, biomechanical, biomarker, and clinical data to support risk stratification and prognostic modeling (32, 33), with potential extension to treatment-response phenotyping. Their clinical translation will require external validation across scanners, acquisition protocols, and populations, transparent reporting of model performance and calibration, and evidence that algorithm-derived features provide additional value beyond established imaging and clinical measures.

### 4.6 Comparison with previous osteoarthritis bibliometric studies

Previous bibliometric studies have examined selected osteoarthritis-related topics, including regenerative treatments and oxidative stress (12, 13). The present study differs in scope and analytical design because it focuses on a specific MRI-defined phenotype that links imaging, clinical symptoms, structural outcomes, subchondral biology, and intervention research.

This study also integrated three bibliographic databases into a deduplicated corpus and combined conventional productivity and collaboration analyses with reference co-citation, harmonized keyword and MeSH analysis, temporal mapping, TF-IDF-based document clustering, and dictionary-based bridge-theme analysis. This multilayered approach was necessary because publication counts and collaboration networks alone could not show how BMLs became connected with pain, progression, whole-joint pathology, biological remodeling, and treatment response. The design is consistent with the broader principle that science mapping is most informative when conceptual, intellectual, and social structures are examined together (35).

## 5 Limitations

Several limitations should also be considered. First, bibliometric analyses depend on database coverage, indexing practices, export quality, and metadata completeness. Although abstracts and cited references were available for nearly all publications, author keywords and MeSH terms were absent for a proportion of the corpus. Second, citation-based measures favor older publications and may underestimate the influence of recent work. Third, affiliation, author, keyword, and cited-reference harmonization required algorithmic and manual decisions, and residual variants may remain.

Fourth, the four temporal periods were selected to represent recognizable phases in the development of the field and were not derived from a formal change-point model. Their interpretation should therefore remain descriptive. Fifth, 2026 was an incomplete publication year. However, exclusion of the 28 publications dated 2026 preserved the overall publication trend, 29 of the 30 leading recent terms, and the principal temporal-theme patterns.

Sixth, the clustering results showed limited separation and should be interpreted cautiously. The slightly negative mean silhouette value (−0.0039) indicates substantial overlap among publications in the standardized TF-IDF space. This overlap may partly reflect the multidisciplinary nature of the literature, because individual publications frequently address several clinical, structural, mechanistic, and methodological themes. Moreover, the cluster-number diagnostics did not converge on a single solution. The two-cluster solution had the highest silhouette width and repeated-subsample stability but produced a broad partition, whereas the gap statistic continued to increase through k = 10, corresponding to a more granular representation. The six-domain solution was therefore retained as an intermediate organizational framework that preserved clinically recognizable research directions while acknowledging uncertainty at domain boundaries. Its mean adjusted Rand index of 0.648 across 20 repeated 80% subsamples, with a minimum of 0.473, indicates moderate reproducibility under resampling; however, this stability does not imply sharply defined or mutually exclusive classes. The six domains should consequently be interpreted as descriptive structures for organizing the literature and not as validated latent categories.

Seventh, dictionary-based bridge-theme mapping improves transparency but remains dependent on the predefined vocabulary and cannot capture every contextual use of a term. Publications could be assigned to multiple themes, and theme prevalence should not be interpreted as a mutually exclusive classification. Finally, terminology surrounding BMLs overlaps with traumatic, postoperative, and non-degenerative marrow abnormalities. The eligibility criteria were designed to retain only studies with an explicit osteoarthritis-relevant component, but some boundary decisions inevitably required judgment.

## 6 Conclusion

MRI-defined BML research in knee osteoarthritis has developed into a multidisciplinary field connecting imaging measurement, pain, structural progression, subchondral remodeling, biomechanics, inflammation, and treatment-oriented investigation. Its intellectual foundation remains anchored by standardized whole-joint MRI assessment and foundational studies of pain and progression, while recent work has expanded toward biological mechanisms, quantitative imaging, and treatment-response phenotyping. The six text-derived domains provide an exploratory organizational framework for this heterogeneous literature and should not be interpreted as discrete biological categories. Future research should prioritize longitudinal, multimodal BML phenotyping that integrates lesion trajectory, quantitative imaging, adjacent joint pathology, biomechanics, subchondral and inflammatory biology, pain mechanisms, and treatment response.

## Data Availability

The original contributions presented in the study are included in the article/Supplementary Material, further inquiries can be directed to the corresponding author.
