# Supplementary material for: From MRI-defined bone marrow lesions to pain, structural progression, and subchondral remodeling in knee osteoarthritis: an integrated multi-database bibliometric and text-mining analysis
Source: Front Med (Lausanne). 2026 Oct 9;13:1903675. doi: 10.3389/fmed.2026.1903675 (PMC13690676; doi:10.3389/fmed.2026.1903675)
Supplement: Supplementary file 1 [file Supplementaryfile2.docx]

**Supplementary Methods**

**Search strategy and study identification**

A comprehensive literature search was performed in Web of Science Core Collection (WoSCC), Scopus, and PubMed to identify publications investigating MRI-defined BMLs or related bone marrow edema-like abnormalities in knee osteoarthritis. The searches were conducted on July 15, 2026, and eligibility was restricted to publications available on or before the prespecified cutoff date of June 1, 2026.

The final strategy was organized around three core concepts: knee osteoarthritis, MRI, and BMLs or bone marrow edema-like lesions. Controlled vocabulary and free-text terms were adapted to the indexing structure of each database. Pain, structural progression, treatment, cartilage loss, joint-space narrowing, and subchondral bone were not required search components. Complete database-specific search strings, field codes, filters, retrieval counts, and export fields are provided in Supplementary Table S1.

The searches initially identified 2,452 records, including 837 from WoSCC, 848 from Scopus, and 767 from PubMed. After the prespecified document-type and language filters were applied where supported, 2,415 records were exported for integration, including 800 from WoSCC, 848 from Scopus, and 767 from PubMed.

WoSCC records were exported as full records with cited references. Scopus records were exported with citation information, abstracts, author and indexed keywords, affiliations, identifiers, cited references, and funding information when available. PubMed records were exported with PMID, DOI, title, abstract, authors, affiliations, journal information, publication type, and MeSH terms. Identification, filtering, deduplication, eligibility, cutoff, and source-overlap counts are provided in Supplementary Table S2.

**Data integration and canonical-record construction**

All exported records were imported into R and converted into a common bibliographic structure. Field names, character encoding, separators, and missing-value representations were standardized before integration. Source-specific identifiers and database membership were retained throughout the procedure.

DOI values were converted to lowercase, URL and prefix components were removed, leading and trailing punctuation was stripped, and blank or invalid values were treated as missing. PMID values were standardized to numeric identifiers. Titles were converted to lowercase, punctuation and repeated whitespace were removed, and typographic variants were normalized.

Duplicate records were identified using a hierarchical procedure. Exact DOI matching was performed first, followed by exact PMID matching and exact normalized-title matching. Remaining unresolved candidate pairs were evaluated using Jaro-Winkler title similarity with a predefined threshold of 0.95 and scaling parameter p=0.1.

A fuzzy-matched pair was accepted only when the normalized title contained at least 30 characters, the first-author surname was compatible, and publication years differed by no more than one year. Exact and accepted fuzzy links were consolidated using connected components to form canonical record groups. Ambiguous pairs were reviewed before merging. The complete deduplication hierarchy and parameters are provided in Supplementary Table S4.

When complementary metadata were available across duplicate records, the canonical record retained the most complete title, abstract, keyword, affiliation, identifier, and cited-reference information. Database-source membership was recorded as a combined source-overlap field.

**Eligibility assessment**

Eligibility assessment was based on the combined title, abstract, keywords, indexing terms, and publication-type information. Full text was consulted when these fields were insufficient for a definitive decision.

Publications were eligible when they addressed knee osteoarthritis, an osteoarthritis-relevant experimental model, or a knee cohort with explicit relevance to osteoarthritis development or progression; involved MRI-defined or imaging-relevant BMLs, bone marrow edema-like lesions, subchondral marrow lesions, or an equivalent explicit construct; and treated BMLs as a substantive exposure, outcome, predictor, imaging phenotype, treatment target, or review topic.

Original research articles and reviews were eligible. Relevant directions included pain, symptoms, function, structural progression, cartilage loss, subchondral remodeling, treatment or intervention, imaging phenotyping, or closely related pathophysiology.

Publications were excluded when they concerned non-knee sites without separable knee data; lacked a knee osteoarthritis or osteoarthritis-relevant context; mentioned BMLs only in background text, indexing terms, adjustment models, or inseparable composite scores; or addressed acute traumatic, postoperative, or other marrow abnormalities without an interpretable osteoarthritis-related component.

Surgical involvement alone did not determine eligibility. Arthroplasty, subchondroplasty, osteotomy, post-traumatic osteoarthritis, and other procedural studies were retained when MRI-defined BMLs contributed to an osteoarthritis-related question concerning symptoms, structural phenotype, progression, or treatment response. Isolated acute injury, reconstruction-related edema, and postoperative marrow changes without osteoarthritis relevance were excluded. Mixed populations were retained only when the osteoarthritis population was dominant or osteoarthritis-specific findings were separately reported. Representative boundary examples are provided in Supplementary Table S3.

**Metadata completeness and entity harmonization**

Metadata completeness was calculated as the number and percentage of final-corpus publications containing non-missing DOI, PMID, abstract, author keywords, indexed keywords or Keywords Plus, MeSH terms, and cited references.

Journal names were standardized using source-title fields and common abbreviation variants. Country or region names were harmonized to standard geographic labels. Institutional names were extracted primarily from complete affiliation blocks. Common abbreviations, parent-system labels, spelling variants, and institutional aliases were consolidated when they referred to the same organization. Isolated generic fragments such as “University,” “Hospital,” or “Department” were removed when they could not be assigned to a specific institution.

Author names were standardized using surname and initials, with database-specific punctuation and spacing variants removed. Conservative harmonization was used to reduce fragmentation without merging authors solely on the basis of a shared surname.

**Keyword and MeSH harmonization**

Author keywords, indexed keywords or Keywords Plus, and MeSH terms were processed separately before combination. Lowercase conversion, punctuation removal, whitespace normalization, singular-plural harmonization, spelling normalization, and abbreviation expansion were applied.

Equivalent expressions were merged into common labels. Examples included knee OA and knee osteoarthritis; MRI and magnetic resonance imaging; bone marrow lesion, bone marrow lesions, and BML; and bone marrow edema and bone marrow oedema.

Generic demographic, indexing, and reporting terms with limited thematic value were removed from co-occurrence visualization. Corpus-defining anchors were retained in descriptive keyword and MeSH analyses because their relative frequencies and connections remained informative. For free-text clustering, highly ubiquitous search-defining anchors were removed to avoid dominance by concepts shared by almost every publication. Examples of merged terms, removed generic terms, and analysis-specific anchor handling are provided in Supplementary Table S5.

**Collaboration analysis**

Country or region, institution, and author networks were constructed using full counting. An entity was represented once per publication, and each pair of entities appearing in the same publication contributed one unit to the corresponding edge weight.

The complete networks were retained for quantitative summaries. For visualization, leading nodes were selected according to publication frequency and network connectivity to preserve legibility. Node size represented publication frequency, and edge width represented collaboration frequency.

At the country or region level, single-country and multi-country publications were calculated separately. A publication was classified as multi-country when at least two distinct countries or regions were represented in the affiliation data. The multi-country publication ratio was calculated as the number of multi-country publications involving an entity divided by its total publication count.

**Cited-reference normalization**

Cited-reference strings were parsed using the available first author, year, source title, volume, page, DOI, and raw-reference text. DOI matching was prioritized when available. Reference labels were standardized to a concise first-author-year-source format for visualization.

Local citation frequency was calculated as the number of final-corpus publications citing each reference. Co-citation edges were generated when two references were cited by the same publication. Edge weights equaled the number of co-citing publications.

The final main co-citation visualization retained 52 valid and highly connected references. Three incomplete author-only strings without identifiable year or source information were excluded. Normalized betweenness centrality was calculated on the final undirected weighted structure to identify references occupying connecting positions between co-citation regions.

**High-intensity local-citation periods**

Annual local citation counts were calculated for each reference using publication year of the citing corpus records. Temporally concentrated influence was summarized using high-intensity local-citation periods derived from the annual citation profiles.

When more than one high-intensity period was identified for the same reference, the period with the greatest strength was retained in the main visualization. Complete period-level outputs were preserved in Supplementary Table S13. The analysis was designed as a transparent corpus-specific measure and was not described as an exact implementation of a proprietary burst-detection algorithm.

**Temporal-period definition**

The four temporal periods were defined as 1996-2008, 2009-2014, 2015-2020, and 2021-2026. The first period represented foundational MRI characterization and early pain, progression, and imaging-pathology research. The second represented expansion of whole-joint MRI cohorts, longitudinal assessment, and semi-quantitative scoring. The third represented methodological and translational maturation, including broader structural and interventional applications. The fourth represented the contemporary phase of mechanistic, quantitative, biomarker, and treatment-oriented research.

The periods contained 48, 162, 257, and 259 publications, respectively. Period boundaries were selected for substantive interpretability and were not derived from a formal statistical change-point model.

**Temporal-theme mapping**

Six non-mutually exclusive temporal themes were evaluated: pain and symptoms; structural progression; subchondral remodeling; synovitis and effusion; treatment and translation; and meniscus and biomechanics.

A publication was considered positive for a temporal theme when at least one predefined expression was detected in the combined title, abstract, author-keyword, indexed-keyword, or MeSH text. Theme prevalence was calculated as the number of positive publications divided by the total number of publications in the corresponding period. A publication could contribute to more than one theme.

The temporal-theme framework used in Figure 5 was distinct from the eight-theme bridge dictionary used for Figure 6. Imaging-defined BMLs and neurovascular mechanisms were not displayed as separate temporal themes in Figure 5 because the former was universal by design and the latter was uncommon during the earliest periods.

**TF-IDF text construction**

Document-level free text was constructed by concatenating titles, abstracts, author keywords, indexed keywords, and MeSH-derived terms. Missing components were represented as empty strings and did not exclude a publication.

Text was converted to lowercase, punctuation and repeated whitespace were normalized, and alphanumeric tokens containing at least three characters were extracted. Standard stopwords and generic biomedical reporting terms were removed. Stemming and lemmatization were not applied to preserve interpretable biomedical terms. Automatic statistical phrase detection was not used. Structured multiword concepts were handled through predefined harmonization before free-text assembly and through explicit dictionary expressions in bridge-theme analyses.

Tokens were retained when they occurred in at least four publications and in no more than 65% of the corpus. Eligible tokens were ordered by document frequency. TF-IDF values were calculated using within-document term frequency and smoothed inverse document frequency. Zero-variance features were removed, and feature columns were standardized before clustering.

**Vocabulary-size selection**

Vocabulary sensitivity was evaluated using the 600, 900, and 1,200 most frequent eligible tokens. The 600-token vocabulary covered 69.2% of cumulative document-frequency mass and 72.2% of token occurrences. The 900-token vocabulary covered 79.0% and 81.1%, respectively. The 1,200-token vocabulary covered 85.6% and 87.1%.

The 900-token specification was selected as an intermediate representation that captured most eligible term use while limiting increasingly sparse and lower-frequency features. Agreement with the 900-token reference clustering was evaluated using adjusted Rand indices.

**Cluster-number evaluation**

K-means clustering was performed for k=2 through k=10 using 80 random starts and a maximum of 200 iterations. Candidate solutions were evaluated using total within-cluster sum of squares, the between-cluster to total sum-of-squares ratio, mean silhouette width, gap statistics, and resampling stability.

Gap statistics were calculated using 30 reference datasets. To reduce computational burden while preserving broad document structure, the gap analysis used the first 100 principal components, which retained 46.9% of the total standardized TF-IDF variance.

Elbow candidates were determined from curvature in the within-cluster sum-of-squares trajectory. Silhouette width was calculated from document distances in the primary feature representation used for the corresponding validation step.

**Resampling stability**

For each candidate value of k, 20 random subsamples containing 80% of the 726 publications were generated without replacement. K-means clustering was repeated within each subsample. All publications were then assigned to the nearest subsample centroid, allowing comparison with the full-corpus solution.

Agreement was quantified using the adjusted Rand index. Mean, standard deviation, and minimum adjusted Rand indices were reported for each candidate k. For k=6, the mean adjusted Rand index was 0.648, with a standard deviation of approximately 0.100 and a minimum of 0.473.

No single validation criterion selected k=6. The two-cluster solution had the highest silhouette width and resampling stability, the gap statistic favored the upper evaluated boundary, and elbow curvature identified several local candidates. The six-domain solution was retained as an interpretability-oriented intermediate level of granularity suitable for organizing the literature. It was not interpreted as a uniquely optimal solution or a naturally separated partition.

**Principal-component visualization**

Principal component analysis was applied to the complete standardized 900-token TF-IDF matrix for visualization. The first principal component explained 1.65% of total variance and the second explained 1.24%. The low combined percentage was expected for a high-dimensional sparse text representation.

The principal-component projection was not used to derive the clusters. Cluster assignments were generated from the complete standardized feature space, and the two-dimensional plot was used only to display approximate document relationships.

**Research-domain labeling**

The six clusters were labeled using three complementary sources: leading terms with high discrimination relative to other clusters, publications nearest each cluster centroid, and bridge-theme prevalence within each cluster.

The final semantic labels were post-traumatic and surgical BML outcomes; community-based structural and risk correlates; subchondral biology, biomarkers, and mechanisms; longitudinal whole-joint progression and symptoms; pharmacologic, biologic, and interventional treatment; and MRI quantification and methodological validation.

The labels describe dominant publication patterns and should not be interpreted as externally validated study classifications or mutually exclusive biological BML phenotypes.

**Bridge-theme dictionary**

Eight bridge themes were defined: imaging-defined BMLs; pain and symptoms; structural progression; treatment and translation; subchondral remodeling; meniscus and biomechanics; synovitis, effusion, and inflammation; and neurovascular mechanisms.

The dictionary was constructed iteratively from established osteoarthritis terminology, representative publications, harmonized structured terms, and discriminating free-text terms. Each concept was represented using explicit words, multiword expressions, abbreviations, and regular-expression variants where appropriate.

Theme matching was performed against the combined title, abstract, author-keyword, indexed-keyword, and MeSH text. A publication was assigned to a theme when at least one dictionary expression matched. Theme assignments were non-mutually exclusive. The complete one-term-per-row dictionary and the corresponding regular expressions are provided in Supplementary Table S9.

**Partial-2026 sensitivity analysis**

The primary corpus included 28 publications dated 2026. To assess the influence of the incomplete publication year, annual trends and recent thematic summaries were recalculated after exclusion of these records, leaving 698 publications through 2025.

Spearman correlation between publication year and annual output was 0.907 when the partial 2026 year was included and 0.946 across complete years through 2025. The corresponding linear slopes were 1.90 and 2.07 publications per year.

Recent-term robustness was evaluated by comparing the 30 leading terms before and after exclusion of 2026. Twenty-nine terms were retained, corresponding to 96.7% overlap. The largest absolute change in recent temporal-theme prevalence was 2.22 percentage points.
