# Supplementary material for: From MRI-defined bone marrow lesions to pain, structural progression, and subchondral remodeling in knee osteoarthritis: an integrated multi-database bibliometric and text-mining analysis
Source: Front Med (Lausanne). 2026 Oct 9;13:1903675. doi: 10.3389/fmed.2026.1903675 (PMC13690676; doi:10.3389/fmed.2026.1903675)
Supplement: Supplementary file 2 [file Supplementaryfile3.docx]

**Supplementary Figure S1. Sensitivity analysis excluding the incomplete 2026 publication year.** Temporal publication patterns, leading recent terms, and temporal-theme prevalence were recalculated after exclusion of the 28 publications dated 2026. The sensitivity analysis included 698 publications through 2025 and was compared with the primary analysis of 726 publications.

**Supplementary Figure S2. Validation of TF-IDF vocabulary size and cluster-number selection.** Complementary diagnostics for candidate solutions from k=2 to k=10 included within-cluster sum of squares, the between-cluster to total sum-of-squares ratio, mean silhouette width, gap statistics, and adjusted Rand indices from 20 repeated 80% document subsamples. Vocabulary sensitivity was evaluated using 600, 900, and 1,200 eligible tokens. No single diagnostic identified a uniquely optimal cluster number; the six-domain solution was retained as an exploratory intermediate-granularity representation.
